# *Ab initio* study of hydrogen and helium diffusion in Be_2_Ti

**DOI:** 10.1039/d5ra01390a

**Published:** 2025-06-03

**Authors:** D. V. Bachurin, C. Stihl, P. V. Vladimirov

**Affiliations:** a Institute for Applied Materials - Applied Materials Physics, Karlsruhe Institute of Technology Hermann-von-Helmholtz-Platz 1 Eggenstein-Leopoldshafen 76344 Germany dmitry.bachurin@kit.edu

## Abstract

Interstitial hydrogen and helium diffusion in the Be_2_Ti compound was investigated *via ab initio* methods. Under certain conditions, this phase can coexist within the desired Be_12_Ti compound, which is a candidate neutron multiplier material for breeder blankets in the DEMO reactor. The Be_2_Ti lattice contains three stable interstitial hydrogen sites and one stable interstitial helium site, all exhibiting lower solution energies than those found in pure beryllium. This indicates a higher solubility of both hydrogen and helium in Be_2_Ti. Diffusion barriers between adjacent hydrogen/helium interstitial sites are calculated using a dimer method. At low concentrations, interstitial hydrogen predominantly diffuses through the energetically favorable interstitial sites A, forming a connected network, with an inter-hexagonal barrier of 0.19 eV. At higher concentrations and elevated temperatures, the diffusion involves less energetically favorable interstitial sites B and C, with higher energy barriers of 0.39 and 0.44 eV, respectively. Interstitial helium diffusion is controlled solely by inter-hexagonal jumps with a barrier of 0.52 eV, while the intra-hexagonal barrier is negligible. The energy barriers between adjacent non-equivalent interstitial hydrogen sites A are at least two times lower than the rate-limiting energy barrier in pure beryllium (0.42 eV), suggesting a higher diffusion rate in Be_2_Ti.

## Abstract 2

We have investigated the migration barriers and diffusion pathways for the interstitial hydrogen and helium diffusion in the Be_2_Ti phase.

## Graphical abstract

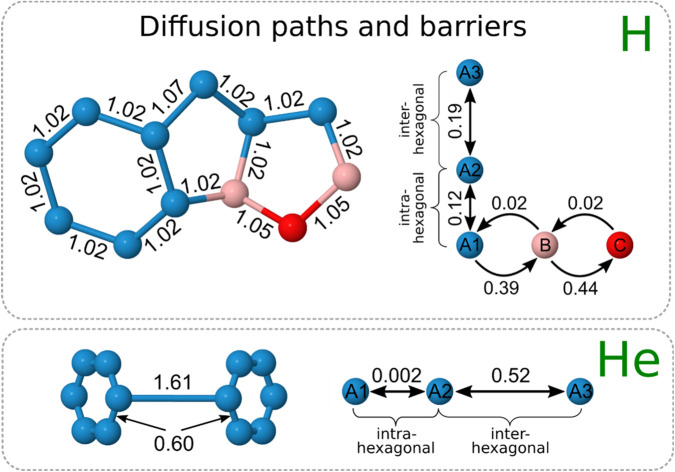

## 1. Introduction

The DEMOnstration Power Plant (DEMO) aims to generate its own fuel, tritium, through the fusion reaction of deuterium and tritium nuclei, producing a neutron and releasing about 14 MeV of energy. The modern tritium blanket design uses prismatic blocks of titanium beryllide as the neutron multiplier material, providing a more cost-effective alternative to the earlier helium-cooled pebble bed (HCPB) blanket design using pure beryllium.^1^ Beryllides are intermetallic compounds formed by alloying beryllium with other elements. Their specific crystal structures and bonding characteristics result in improved mechanical properties such as strength, hardness, improved high-temperature stability and oxidation resistance, lower tritium retention and swelling, higher melting point and good compatibility with structural materials.

Under neutron irradiation, besides the formation of interstitials, vacancies and their clusters, beryllium atoms undergo transmutation into tritium and helium through nuclear reactions. At elevated temperatures tritium diffuses and can be captured by existing defects, leading to the formation of gas bubbles. The concentration of tritium accumulated within beryllide is much lower than that of helium, so tritium does not significantly affect the microstructure. However, radioactivity of tritium is a major concern. Therefore, after a nuclear reactor's operation is complete, the decommissioning of breeding blanket modules (beryllide blocks or pebbles) requiring proper handling of radioactive wastes is an important task. As a result, estimating the tritium retention in beryllides becomes a priority.

One possible solution to simplify the recycling of hundreds of tons of beryllides required for the operation of the DEMO reactor is to facilitate tritium release. Extensive research on beryllides for fusion applications started recently. The preparation methods and physical properties of titanium beryllides were studied in ref. 2–15. Experimental studies on thermal desorption have revealed that deuterium is released from Be_12_Ti at lower temperatures than from pure beryllium.^5,16,17^ The Be_12_Ti phase is desired, but other phases such as Be_2_Ti, Be_17_Ti_2_, pure beryllium, and titanium are formed and present in the structure under certain conditions.^7,8,18,19^ Therefore, hydrogen and helium will need to migrate through all these phases upon release from traps. To assess tritium retention, it is necessary to study the diffusion paths and characteristics of all phases involved.

It may turn out that in one of the phases the diffusion barriers will be significantly higher than in others. Therefore, the presence of such intermetallic phase will prevent the early release of tritium from the material, ultimately increasing its retention. Presently, researchers are primarily focused on studying one phase of titanium beryllide, specifically Be_12_Ti. *Ab initio* modeling revealed that the early release of tritium can be attributed to the lower binding energy of hydrogen with vacancies compared to pure beryllium.^20,21^ Additionally, it was discovered that in Be_12_Ti, three main hydrogen diffusion paths can be distinguished along the *a* and *c* crystal axes.^22^ At the same time, practically no attention was paid to other phases that, under certain conditions, can coexist with the main phase. Although the volume fraction of these additional phases may be small, it is still important to study potential hydrogen and helium diffusion paths within them. This is necessary for calculating the diffusion coefficient, which is one of the crucial parameters in assessing tritium retention.

The aim of this work is to investigate the migration barriers and diffusion pathways for the interstitial diffusion of hydrogen and helium in the Be_2_Ti phase using the *ab initio* methods. The choice of Be_2_Ti phase is motivated by its inevitable presence in the material and its significance in understanding fundamental diffusion mechanisms before extending the study to more complex phases such as Be_17_Ti_2_. While Be_17_Ti_2_ may indeed provide additional interstitial sites and migration pathways, adopting a stepwise approach - starting with Be_2_Ti - allows us to establish reliable data for evaluating diffusion coefficients, which can serve as a reference for more intricate systems.

Despite the high levels of displacements per atom during irradiation, the overall concentration of point defects remains relatively low due to mutual recombination and annealing processes. At such low defect concentrations, understanding intrinsic diffusion in an ideal crystal lattice becomes crucial, as the effects of trapping by vacancies or self-interstitial atoms can be evaluated using chemical rate theory or cluster dynamics approaches. Both methods require accurate diffusion coefficients as input parameters, underscoring the importance of the present study.

## 2. Modeling technique

The Vienna *Ab initio* Simulation Package (VASP) is used for all density functional theory (DFT) computations. Projector-augmented wave pseudopotentials^23^ are employed with a generalized gradient approximation^24^ for the exchange-correlation functional. The pseudopotentials for beryllium (with two valence electrons), titanium (four electrons), hydrogen (one electron), and helium (two electrons) are sourced from the VASP library. Spin polarization was initially tested through calculations starting from antiparallel and parallel initial magnetic moments and were generally found to ultimately converge in the same magnetic configuration (for more details, see our previous publications^21,25^). Thus, subsequent calculations, such as saddle point searches, were carried out starting from parallel initial magnetizations only. The structural model of the selected titanium beryllide was obtained from the materials project,^26^ a collaborative online platform that provides a wealth of data related to the properties of inorganic materials.

The Brillouin zone sampling is carried out using an automatically generated grid with minimal distances of 0.12 Å^−1^ (KSPACING-tag). The plane-wave basis set cutoff energy is chosen to be equal to 487 eV (ENCUT-tag). Electronic and ionic convergence are assumed when the difference in total energy between subsequent electronic iterations is less than 1.0 × 10^−5^ eV and all residual atomic forces are less than 5.0 × 10^−3^ eV Å^−1^ (EDIFF- and EDIFFG-tags), respectively. A second-order Methfessel–Paxton scheme with a width of 0.2 eV (ISMEAR- and SIGMA-tags) is used to smooth the step at the Fermi level, resulting in negligible entropy terms of approximately 1.0 meV per atom. Further information on the precise parameter selection process can be found in the Appendix and in the Computational Technique section of our previous publication.^21^ The volume and shape of the simulation cell remain unchanged throughout all calculations and there are no constraints on the movement of the atoms. Periodic boundary conditions are implemented along all three crystallographic axes. Atomic structures are visualized using the open-source molecular viewer Jmol.^27^

The crystal structure of Be_2_Ti (the space group of *Fd*3̄*m*) accommodates one symmetrically non-equivalent beryllium site Be1[16d] (0.375, 0.125, 0.825) and one symmetrically non-equivalent titanium site Ti1[8a] (0.75, 0.75, 0.25). The Wyckoff symbols are given in square brackets and the position vectors in terms of lattice vectors are provided in parenthesis. The primitive unit cell of the Be_2_Ti has edges *a* = *b* = *c =* 4.54 Å and angles *α* = *β* = *γ* = 60°, while the conventional cell has edges *a* = *b* = *c =* 6.43 Å and angles *α* = *β* = *γ* = 90°. The simulation cell throughout all calculations consists of 2 × 2 × 2 unit cells (48 atoms) of Be_2_Ti and an additional interstitial (hydrogen or helium) atom at respective sites of interest.

A separate convergence test confirmed that the chosen simulation cell size is adequate: increasing the cell to 3 × 3 × 3 unit cells (162 atoms) changed the solution and migration energies by no more than 0.01 eV. This demonstrates that the selected size meets the desired level of accuracy.

The modelling process can be divided into two parts. Firstly, the stable interstitial sites of atomic hydrogen and helium in the crystal lattice of Be_2_Ti are identified. Secondly, the energy barriers associated with atomic diffusion processes between these stable interstitial sites are calculated. Both parts are implemented as follows.

(i) Candidate interstitial sites for hydrogen and helium are identified as the nodes of a Voronoi tessellation of the Be_2_Ti structure. Voronoi vertices are equidistant from four neighbor atoms pointing to possible interstitial hole positions. We used Voronoi construction as implemented in the Pymatgen code, providing a wide range of tools for generating, analyzing, and modifying crystal structures.^28^ When testing each of these candidate sites for stability employing structural optimization with VASP, all configurations are perturbed by displacing all atoms by 0.01 Å from their equilibrium lattice positions in a random direction. This is done to avoid erroneous convergence due to retained symmetries resulting in force cancellation. The sites of hydrogen or helium from all these structural optimizations are stable interstitial sites. In general, such stable interstitial sites are routinely found to be unphysically close to stable sites found in earlier calculations or one of their symmetrically equivalent sites. Therefore, only sites located at a distance greater than 0.1 Å are considered to be non-equivalent. Further details of this approach can be found in ref. 21.

(ii) To calculate the energy barriers associated with diffusion processes, the dimer method has been adopted.^29–31^ It considers a pair of two configurations, the dimer, separated by the total dimer length 5.0 × 10^−3^ Å. Employing repeated translation of the dimer according to an effective translational force with inversed parallel components and rotating into the direction of minimum curvature, the dimer center eventually converges in a saddle point configuration. This method is implemented in the VASP Transitional State Theory (VTST) package,^32^ and is often used as a computationally less expensive alternative to the Nudged Elastic Band (NEB) method^33,34^ to find a nearby saddle point in a given direction, starting from an initial configuration close to the initial minimum configuration, *i.e.* a stable interstitial site. The use of the dimer method requires considerably lower electronic convergence thresholds of about EDIFF = 10^−7^, to facilitate the finite difference evaluations during rotational iterations, while the other parameters remain unchanged. For more details on how the following dimer calculations were set up, the reader is referred to the VTST website^32^ and our previous publication.^22^

During the extensive diffusion barrier search campaigns presented in this work, a purely geometric surrogate parameter was identified to indicate particularly relevant potential diffusion jumps. This parameter, called the “diffusive solid angle”, clearly shows a massive drop after its four highest values, delimiting the exact cases where no new diffusion jumps were found anymore. To compute the diffusive solid angle, the ideal crystal structure is augmented by all stable interstitial sites, *i.e.* A, B, and C, as well as their symmetric equivalents. By performing a Voronoi tessellation of this augmented structure, Voronoi pairs of sites sharing a common facet of the Voronoi polyhedra surrounding them are identified. If both sites of such a Voronoi pair are interstitial sites for hydrogen or helium, the solid angle concealing their common facet contributes a diffusive solid angle. This parameter is directly proportional to the area of a ring of atoms through which hydrogen is jumping and inversely proportional to the jump length. It is quite useful in prioritizing saddle point search calculations and determining whether a given calculation is likely to be worth the computational effort in terms of discovering a new diffusion jump.

## 3. Results and discussion

### 3.1. Stable interstitial sites for hydrogen and helium

Diffusion of hydrogen and helium in crystal lattices is assumed to occur through the interstitial mechanism. This is the fastest way of diffusion which implies that these atoms can diffuse through the crystal lattice by hopping from one interstitial site to another. As a prerequisite of the subsequent study of diffusion paths, it is essential to identify all stable non-equivalent interstitial sites for hydrogen and helium atoms in the crystal lattice as described in the Modeling Technique section above.

Solution energy is a measure of the energetic preference of a particular interstitial site and can be calculated using the following formula:

1 *E*^X^_s_ = *E*^X+H/He^ − *E*^X^ − *E*^H/He^_ref_,

where *E*^X+H/H*e*^ and *E*^X^ (X = Be, Be_2_Ti) are the total energies of the simulation cell with and without interstitial hydrogen/helium, respectively. The reference energy of a hydrogen atom was calculated as half of the energy of a hydrogen molecule, 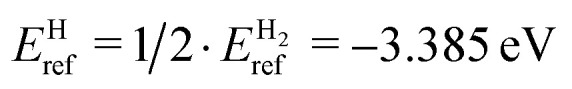, while the reference energy of a helium atom, *E*^He^_ref_ = −0.001 eV, was obtained from a single helium atom in a simulation cell. The sizes of both computational cells were chosen to minimize spurious interactions with periodic images. The calculated reference energies of individual hydrogen and helium atoms were found to be in good agreement with the results reported by other authors.^21,25,35–41^

Static *ab initio* calculations reveal three stable symmetrically non-equivalent interstitial hydrogen sites and only one stable symmetrically non-equivalent interstitial helium site in Be_2_Ti. All other interstitial sites for hydrogen or helium within the crystal were found to be unstable, *i.e.* they converge to one of the respective stable sites or one of their symmetrical equivalents. All unstable interstitial sites were discarded. Interstitial diffusion occurs only between the stable interstitial sites. The remaining stable sites are labeled with the capital letters A, B, and C in order of increasing solution energy. For convenience, all the results are summarized in Table 1. Note that hydrogen and helium interstitial sites sharing a common label do not imply any further similarity, the labels are simply assigned to refer consistently between symmetrically non-equivalent sites throughout the paper.

**Table 1:** Non-equivalent stable interstitial sites for a single hydrogen/helium atom in Be_2_Ti, sorted in order of increasing solution energy, *E*_s_ (in eV). The abbreviations in brackets refer to basal-split mixed (BS_m_) dumbbell, basal tetrahedral (BT), and basal octahedral (BO). A dash in a column indicates that this stable interstitial site does not exist

Material	A	B	C	D	E	F	G
Be_2_Ti + H	0.01	0.38	0.79	—	—	—	—
Be_2_Ti + He	3.31	—	—	—	—	—	—
Be + H^21^	1.50 (BT)	1.70 (O)	—	—	—	—	—
Be + H^43^	1.58 (BT)	1.79 (O)	—	—	—	—	—
Be + H^39^	1.48 (BT)	1.68 (O)	—	—	—	—	—
Be + H^38^	1.40 (BT)	1.61 (O)	—	—	—	—	—
Be + He^25^	5.45 (BS_m_)	5.81 (BO)	—	—	—	—	—
Be + He^44^	5.39 (BS_m_)	5.70 (BT)	—	—	—	—	—
Be + He^39^	5.62 (BT)	5.71 (BO)	—	—	—	—	—
Be + He^38^	5.43 (BT)	5.64 (BO)	—	—	—	—	—
Be_12_Ti + H^21^	0.50	0.73	0.84	0.90	0.93	0.98	1.27
Be_12_Ti + H^41^	0.50	0.80	0.92	0.94	0.98	1.20	1.30
Be_12_Ti + He^25^	4.14	4.16	4.27	4.63	—	—	—
Be_12_Ti + He^41^	4.03	4.38	4.53	4.87	—	—	—

**Fig. 1:**
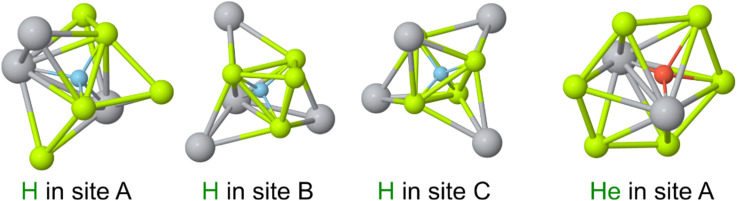
Stable interstitial hydrogen and helium sites within the crystal lattice of Be_2_Ti. Only beryllium and titanium atoms located within a 2.90 Å radius from the interstitial are displayed. Interstitial hydrogen and helium atoms are connected to the nearest beryllium and titanium atoms by thin lines to indicate their precise positions in the lattice.

Three non-equivalent stable interstitial sites were identified for hydrogen in Be_2_Ti with solution energies ranging from 0.01 to 0.79 eV (see Fig. 1). The site A with the lowest solution energy is located within a tetrahedron formed by two Be1[16d] atoms and two Ti1[8a] atoms. This suggests that hydrogen will predominantly occupy interstitial site A at any temperature in equilibrium. As demonstrated in ref. 42, this solution energy may even be negative, implying an endothermal hydrogen occlusion from gas phase. The interstitial site B is situated within a tetrahedron formed by three Be1[16d] atoms and one Ti1[8a] atom. The site C is located at the center of a tetrahedron formed by four Be1[16d] atoms and has the highest solution energy among the identified stable interstitial sites of hydrogen.

The crystal lattice of Be_2_Ti has only one stable interstitial helium site located in the tetrahedron formed by two Be1[16d] atoms and two Ti1[8a] atoms as illustrated in Fig. 1. This is the same location as the interstitial site A for hydrogen. However, the helium atom is positioned off-center within the tetrahedron and is near the imaginary line connecting the two Ti1[8a] atoms. The helium atom at this position has a solution energy of 3.31 eV, which is significantly higher than that of a hydrogen atom in any of its stable interstitial sites. To date, no other publications except^21^ have addressed the study of stable interstitial hydrogen/helium sites in Be_2_Ti to our knowledge.

### 3.2. Diffusion barriers and diffusion paths

The following analysis considers the diffusion of hydrogen and helium atoms in Be_2_Ti, assuming their movement between two stable adjacent interstitial sites separated by a migration energy barrier. Further, the diffusion of hydrogen and helium will be considered in different subsections. The lattice of Be_2_Ti enables various jumps between equivalent interstitial sites that differ in both jump directions and distances. To avoid confusion, these stable sites are labeled with appended numbers, such as A1, A2, *etc.*

#### 3.2.1 Hydrogen

**Fig. 2:**
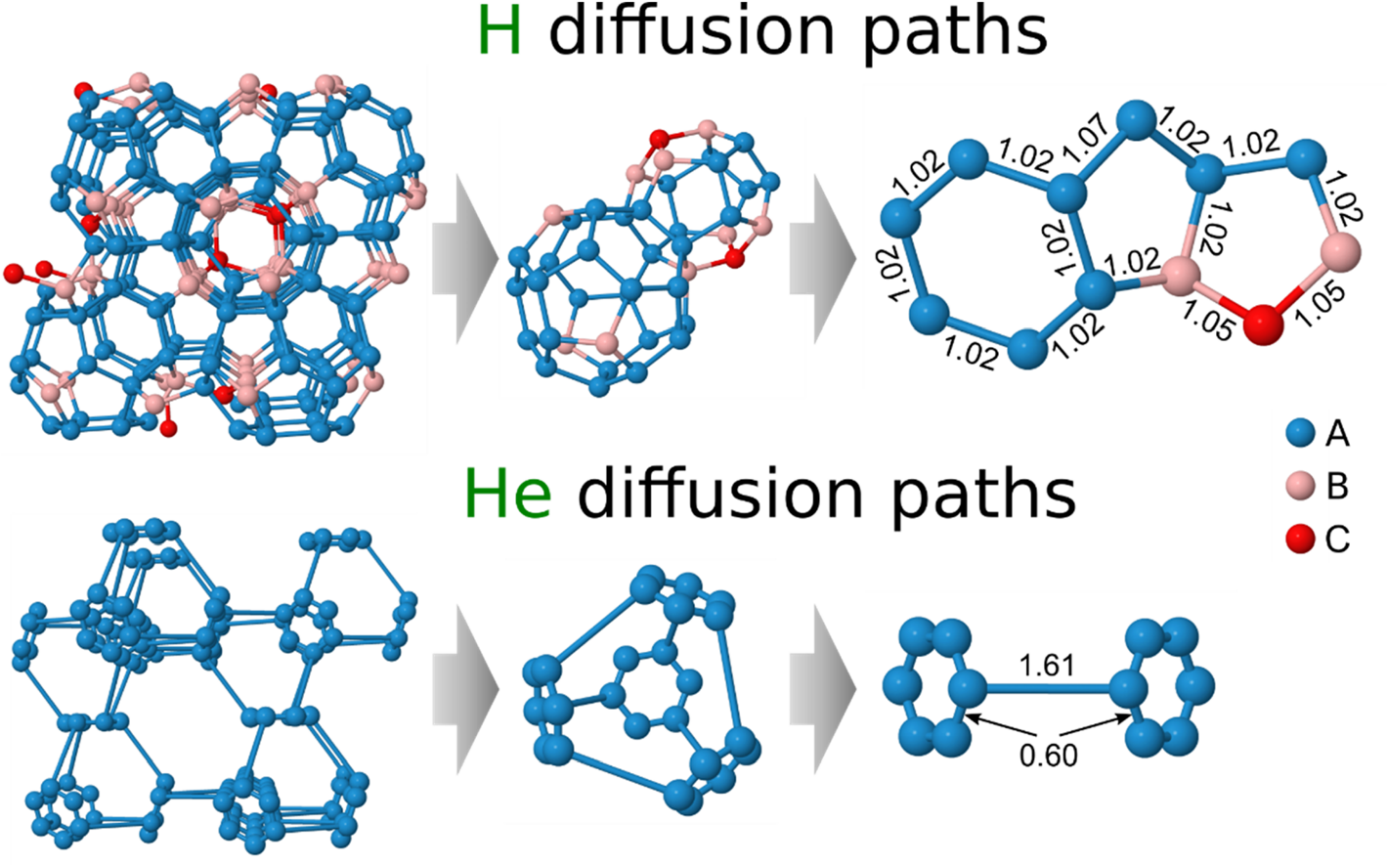
The stable interstitial hydrogen (top) and helium (bottom) sites within the crystal lattice of Be_2_Ti. The main diffusion path patterns formed by regular polygons, along with all non-equivalent polygons forming polyhedra, are presented. Bonds between neighboring stable interstitial sites illustrate possible diffusion jumps (in Å).

Fig. 2 illustrates all stable interstitial sites and diffusion paths for hydrogen within the crystal lattice of Be_2_Ti. To provide clarity and avoid cluttering the figure, beryllium and titanium atoms are not shown. The stable interstitial sites are color-coded based on their solution energy, as indicated in Table 1. The left panel of the figure demonstrates all stable equivalent interstitial sites along which diffusion can occur in the lattice. The middle panel highlights primary patterns formed from regular polygons. On the right, all feasible polygons and non-equivalent jumps between the interstitial sites are depicted. Only nearest neighboring interstitial sites are connected by bonds to indicate potential diffusion paths.

From the rather complex network of hydrogen diffusion paths, two main polyhedrons composed of interstitial sites can be distinguished. The first polyhedron comprises interstitial hydrogen sites A and B located around the titanium atom as shown in the middle panel of Fig. 2. This polyhedron is composed of four hexagons containing only interstitial sites A, with a distance of 1.02 Å between them, and twelve pentagons consisting of interstitial sites A and B with the same distance of 1.02 Å between all sites, except for two sites A that are spaced 1.07 Å apart (see right panel). The second polyhedron comprises six pentagons that contain one C site, two A and two B sites, as well as six pentagons that contain four A and one B sites. The distance between stable interstitial sites B and C is 1.05 Å. Thus, the following non-equivalent jumps can be distinguished between the stable hydrogen sites in Be_2_Ti, including two A–A jumps that differ in distance from each other, as well as A–B and B–C jumps.

**Fig. 3:**
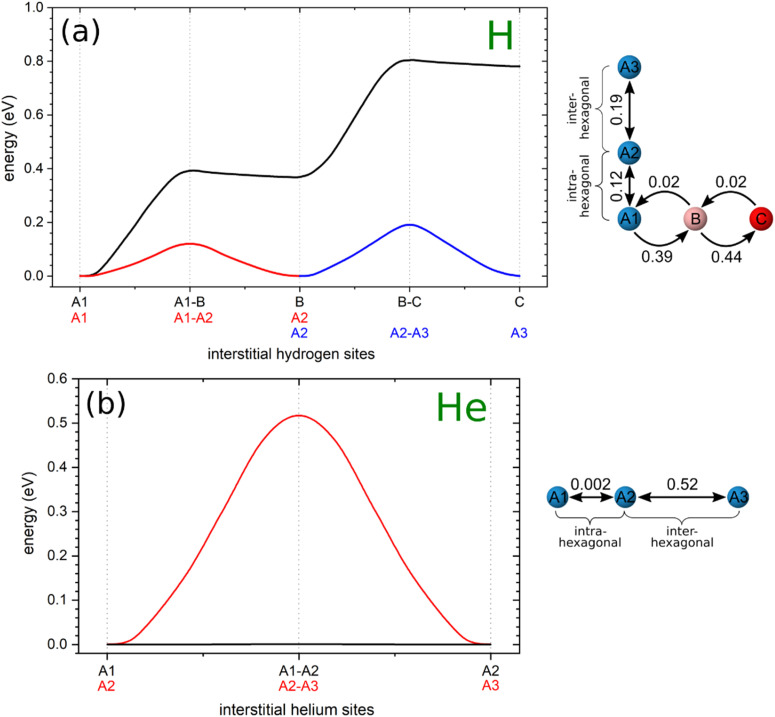
The energy barriers between adjacent non-equivalent interstitial sites for (a) hydrogen and (b) helium jumps along the selected diffusion paths. The arrow indicates the direction of the jump and the value represents the height of the diffusion barrier (in eV). Hydrogen and helium atoms are color-coded based on their solution energy (see Table 1 and Fig. 2). The reference point, set as zero, corresponds to the minimal solution energy at site A. The lines connecting stable and saddle points serve as guides to the eye.

Fig. 3a displays the energy landscape for interstitial hydrogen diffusion in Be_2_Ti. The energy barriers for the intra-hexagonal A1–A2 and inter-hexagonal A2–A3 jumps are 0.12 and 0.19 eV, respectively, which are significantly smaller than for the A–B (0.39 eV) and B–C (0.44 eV) barriers. Therefore, at low temperatures and low concentrations hydrogen tend to accumulate and diffuse through the energetically favorable interstitial sites A, which form a connected network allowing jumps in all directions even without involving interstitial sites B and C.

To enable A → B and B → C jumps, hydrogen must overcome energy barriers of 0.39 and 0.44 eV, respectively. The heights of the reverse B → A and C → B jumps are only 0.02 eV, which makes the sites B and C weakly stable for interstitial hydrogen. As a result, they are unlikely to be occupied if there are empty sites A in the lattice. Therefore, at low concentrations and low temperatures, hydrogen diffuses preferentially along the energetically favorable sites A. Furthermore, this phenomenon occurs over a wide range of concentrations since the crystal lattice of Be_2_Ti has a significantly higher number of sites A (≈70.5%) compared to sites B (≈23.5%) and C (≈6.0%). The jump network connecting sites A with a barrier of 0.19 eV represents the minimum-energy pathway for interstitial hydrogen diffusion, controlling long-distance transport in all crystallographic directions. As the concentration and temperature increase, the interstitial sites B become occupied, followed by the sites C.

#### 3.2.2 Helium

The diffusion paths formed from the only stable interstitial helium site A in Be_2_Ti are shown in Fig. 2. The main element of the diffusion network for interstitial helium is a truncated tetrahedron, which includes two diffusion jumps: intra-hexagonal occurring between stable helium sites within the same hexagon with a length of 0.60 Å, and inter-hexagonal, connecting two different hexagons with a length of 1.61 Å. The interstitial helium diffusion in Be_2_Ti is restricted by the inter-hexagonal jumps with a barrier of 0.52 eV, which allows helium to migrate between hexagons and thus travel through the crystal. Before the onset of this diffusion, helium is found to diffuse intra-hexagonally with a negligible barrier of 0.002 eV.

**Fig. 4:**
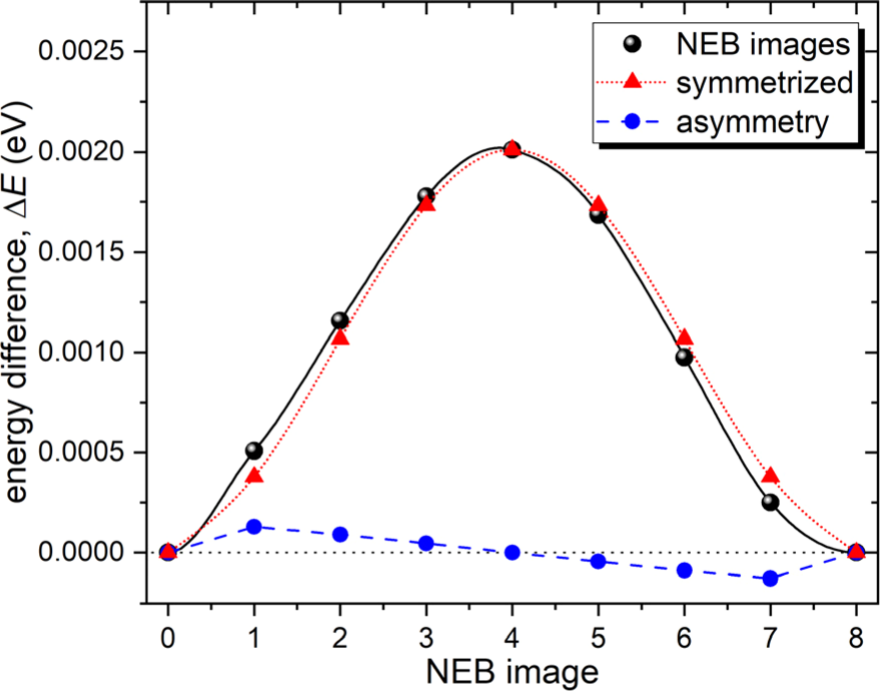
The energy barrier between adjacent non-equivalent interstitial helium sites A for intra-hexagonal jump calculated using NEB method. For comparison, a symmetrized jump and the corresponding asymmetry are also presented. Horizontal dotted line and the solid lines connecting the data points serve as a guide to the eye.

This extraordinarily small barrier prompted additional stability tests. To ensure the existence of a hexagonal configuration formed by stable interstitial sites, a structural optimization with a lowered convergence threshold of residual forces was performed from the previously converged interstitial structure. As a result, the interstitial sites did not move noticeably towards the center of the hexagon, thus confirming the stability of the hexagonal configuration. In contrast to all other barriers, the dimer method failed to converge to the intra-hexagonal barrier configuration. Instead, the computationally more demanding nudged elastic band (NEB) approach,^45^ as implemented in VTST, was successful in finding the barrier. The resulting barrier profile for the intra-hexagonal jump with seven intermediate images between the initial and final minima is demonstrated in Fig. 4. Note that although the real barrier must be perfectly symmetric due to crystal symmetries, there are tiny asymmetries of about 10^−4^ eV. These can be explained by the residual asymmetries introduced by small random shifts to avoid force cancellation during the structural optimizations as described in the Modeling Technique section. Such an extremely low barrier value indicates that in reality, no barrier exists, and helium can freely move along these six sites A forming the hexagon. The latter suggests that hydrogen is trapped near a titanium atom, resulting in rapid movement around a single point that does not contribute to the net mass transfer as measured by diffusion. This effect is well known for other materials and is commonly referred to as caging or trapping.^46^ Thus interstitial helium diffusion in Be_2_Ti is primarily controlled by inter-hexagonal jumps with a barrier of 0.52 eV connecting the surrounding titanium atoms.

## 4. Discussion

The solution energy gives an indication of the thermal equilibrium concentration of gases in the material and serves as a measure of solubility. The excessively high solution energy of helium confirms its insolubility in Be–Ti intermetallics and reinforces the understanding that helium cannot be thermally introduced, but rather forms bubbles upon nuclear transmutation. Furthermore, the comparison of solution energies allows for the assessment of the relative affinity of different Be–Ti phases for hydrogen and helium. Diffusion barriers, on the other hand, determine the mobility of these gases within the material. Low migration barriers along diffusion path suggest rapid transport along this path, while higher barriers indicate that gas atoms are more likely to be trapped inside crystal lattice, affecting their long-term retention and potential bubble formation. There are currently no other publications devoted to the study of the interstitial hydrogen and helium diffusion in the Be_2_Ti compound. Therefore, below we will compare our results with those obtained earlier by other authors for the Be_12_Ti and pure beryllium.

The authors^22^ considered the interstitial hydrogen diffusion in the Be_12_Ti and found that the height of the diffusion barriers varies between 0.02-0.57 eV, which is significantly higher than the intra-hexagonal and inter-hexagonal jumps (0.12 and 0.19 eV) in Be_2_Ti (see Fig. 3a). Zhu and co-authors^41^ have calculated only three possible diffusion paths in the Be_12_Ti lattice using first-principles calculations and the climbing nudged elastic band method. Two of them seem to be irrelevant for the comparison, since they were calculated between the unstable hydrogen sites. However, the third energy path A → C (0.45 eV) and C → A (0.15 eV) agrees well with the results of ref. 22. Thus, from the comparison of the above results for interstitial hydrogen diffusion, it can be concluded that when the Be_2_Ti and Be_12_Ti phases coexist as demonstrated in ref. 7, 8, 18 and 19, at low temperature, the hydrogen diffusion occurs predominantly in the Be_2_Ti phase and only at elevated temperature it begins to be activated in the Be_12_Ti phase.

A single energy barrier for interstitial helium diffusion in the Be_12_Ti phase is given, which is found to be 0.35 eV in ref. 41. The energy barrier controlling diffusion in the Be_2_Ti phase found in the present work is slightly higher (0.52 eV), suggesting a lower helium diffusion rate. Such a comparison is very superficial, and for a final answer to the question of the rate of diffusion processes, it is necessary to make a complete study of all diffusion barriers for helium in the Be_12_Ti phase.

The available results related to hydrogen and helium diffusion in pure beryllium are presented below. Beryllium has two stable hydrogen interstitial sites, namely basal tetrahedral (BT) and octahedral (O),^21,40^ and two stable helium interstitial sites, which are the basal-split mixed dumbbell (BS_m_) and basal octahedral (BO).^44^ According to Zhang *et al.*,^37^ the dominant diffusion mechanism for interstitial hydrogen in pure beryllium is the BT → O → BT path with an associated energy barrier of 0.40 eV. Very similar values for this diffusion path have been reported in ref. 43 and 47. The energy barriers for interstitial helium diffusion are equal to 0.14 and 0.06 eV along the BT → BO → BT path. These are significantly lower than the energy barriers for interstitial hydrogen diffusion.^37^ Ganchenkova *et al.*^40^ have found that the same diffusion path is the most energetically favorable with a barrier of about 0.10 eV. Thus, in contrast to hydrogen, helium has a relatively low migration barrier and as a consequence higher diffusion rate in pure beryllium, which is in good agreement with results on helium diffusion in other metals.^48–50^

It is worth noting that the authors^37^ did not find the most energetically favorable interstitial helium site in pure beryllium, namely the mixed dumbbell, and therefore consider diffusion jumps between the second most favorable BT sites. As shown in ref. 44, the difference in solution energies between the mixed dumbbell and BT sites depends on the size of the computational cell and is of the order of 0.30 eV.

## 5. Conclusions

*Ab initio* methods were used to investigate interstitial hydrogen and helium diffusion in Be_2_Ti compound. The outcomes yield the following conclusions.

The lattice of Be_2_Ti contains three stable interstitial hydrogen sites and one stable interstitial helium site. These sites have lower solution energies than those found in pure beryllium, indicating higher solubility of both hydrogen and helium in Be_2_Ti.

At low concentrations, interstitial hydrogen diffuses predominantly through the interstitial sites A, forming a connected network, and requires overcoming an inter-hexagonal barrier of 0.19 eV. At high concentrations and elevated temperatures, the diffusion process involves interstitial sites B and C, which require overcoming higher energy barriers of 0.39 and 0.44 eV, respectively.

Interstitial helium diffusion is controlled only by inter-hexagonal jumps with barriers of 0.52 eV. The height of the intra-hexagonal barrier is negligible.

The energy barriers between adjacent non-equivalent interstitial hydrogen sites A required to initiate the diffusion process are at least two times lower than the rate-limiting energy barrier in pure beryllium (0.42 eV), suggesting a higher diffusion rate in Be_2_Ti.

The obtained barrier heights serve as essential input for future calculations of the diffusion coefficients of hydrogen and helium in Be_2_Ti phase. Such calculation is particularly important for the evaluation of tritium retention in beryllium blocks, which are planned for use as neutron multipliers in the DEMO reactor.

## Data availability

The raw/processed data required to reproduce these findings cannot be shared at this time as the data was obtained within the frames of the EUROfusion project.

## Conflicts of interest

There are no conflicts to declare.
